# Matters of Medical Gossip

**Published:** 1856-02

**Authors:** 


*Matters of Medical Gossip.*—The wars are all over—New York has given up the discussion of catheterization of the air passages, and Prof. Green introduces his probang without further opposition from the profession, whatever obstacles he may meet at the chordæ vocales. Philadelphia has, with a single indignant grunt, settled down in resignation to the appointment of Prof. Smith to the chair of Physic and Gibson. The New Orleans *savans* for once agree that a rigid quarantine cannot do away with the effect of tropical heat and humidity upon the filth of an uncleanly city, and we have even reason to suppose that some of them no longer insist so strongly on the prophylactic qualities of decaying organic matter. At Washington, the Smithsonian Institution continues its sleepy dream about "diffusing knowledge among *men*"—that is the emphasis they put upon it, as if to exclude women and all other mammalia, whether ruminants, ungulata, or pachyderms—while Prof. Blodget finds refuge from its inhospitable walls in the Surgeon General's office, and there, in a few months, elaborates a work upon climate, which leaves nothing but a beaten track for the stupid old government school to follow in. At Boston they succeed in maintaining their modern Athenian dignity; and at Albany they are in a "dwam" whether people die soonest from ether, chloroform, air in the jugular, blood out of it, or severance of the thoracic duct (!) At Plattsburg, the Northern Lancet, once belligerent beyond measure, the doughty opponent of Dr. Reese, and the chivalric supporter of Prof. Bedford, has dwindled to an emaciated weekly, published on the dirtiest of whitey-brown paper, as meek as Moses in its editorials—and so the wars are all over.

Not quite. Our quondam antagonist, the Peninsular Journal, with its four capital editors, wages bloody contest on Dr. Davis of the Northwestern. "The young man from Binghampton," replies with spirit, pitches into Government Schools generally, discourses on the anæsthetic influence of government pap in its operation on salaried professors, and recites with vigorous unction those manifold arguments which erst we uttered in our own little quarrel with the same party some years agone. Ah! Dr. Davis! we would like to help you in that battle, and did you need us, you would find us at your side *instanter*.

Our own pet discussion on pneumonia is not dead yet—indeed the heaviest gun yet fired graces our present issue. In the meantime a side-fight is going on in private and hospitable practice, and the ides of March will enable the "tendency-to-recovery-party" to enumerate cases by the dozens and fifties, of double, single, sthenic, asthenic, simple and complicated pneumonia, going on to health under the mildest treatment.

These latter however are, we believe, the only belligerencies now manifest in the Journals. Let us look for news at home and abroad.

